# Data Mechanics and Coupling Geometry on Binary Bipartite Networks

**DOI:** 10.1371/journal.pone.0106154

**Published:** 2014-08-29

**Authors:** Hsieh Fushing, Chen Chen

**Affiliations:** Department of Statistics, University of California Davis, Davis, California, United States of America; National Institute of Genomic Medicine, Mexico

## Abstract

We quantify the notion of pattern and formalize the process of pattern discovery under the framework of binary bipartite networks. Patterns of particular focus are interrelated global interactions between clusters on its row and column axes. A binary bipartite network is built into a thermodynamic system embracing all up-and-down spin configurations defined by product-permutations on rows and columns. This system is equipped with its ferromagnetic energy ground state under Ising model potential. Such a ground state, also called a macrostate, is postulated to congregate all patterns of interest embedded within the network data in a multiscale fashion. A new computing paradigm for indirect searching for such a macrostate, called Data Mechanics, is devised by iteratively building a surrogate geometric system with a pair of nearly optimal marginal ultrametrics on row and column spaces. The coupling measure minimizing the Gromov-Wasserstein distance of these two marginal geometries is also seen to be in the vicinity of the macrostate. This resultant coupling geometry reveals multiscale block pattern information that characterizes multiple layers of interacting relationships between clusters on row and on column axes. It is the nonparametric information content of a binary bipartite network. This coupling geometry is then demonstrated to shed new light and bring resolution to interaction issues in community ecology and in gene-content-based phylogenetics. Its implied global inferences are expected to have high potential in many scientific areas.

## Introduction

A binary matrix is commonly used to record a collection of indices for the presence/absence of dyadic relations between two non-ordinal categorical variables of interest: one has all its nodes listed on the rows, the other on the columns. This data type can be traced back to Darwin's (1839) *The Voyage of The Beagle*, in which he reported the famous “Darwin's Finches”data. This kind of data matrix is also called a co-occurrence matrix, or a binary contingency table [1], and is popularly represented via a binary bipartite graph (or network) in the context of Graph theory [2], [3].

Such a data type becomes the center of controversy in community ecology right after Jared M. Diamond proposed “community assembly rules” [4]. While this data type has been continuously collected (e.g., bird vs. island [5], lizard vs. island [6], plant vs. niche [7], [8] and many others [9]), community ecologists have continued debating about the two mechanistic rules comprising natural selection forces: 1) Forbidden species random combinations; and 2) Reduced niche overlap, for the last five decades [10]–[14]. At this point in time, it might be right to ask a neutral, but fundamental question: what are the global features or nonparametric information contents contained within such a binary matrix? In our opinion, the controversy is primarily caused by missing proper answers to this question.

On another front of science in the last decade, the advent of genome sequencing has led to the building of species phylogeny based on the whole gene content, instead of on comparisons between single genes [15]. Ideally this gene content based phylogeny could be less sensitive to inconsistencies due to horizontal gene transfer, unrecognized paralogy and highly variable rates of evolution [16], [17]. This important development in phylogenetics also relies on binary bipartite network data. However, its goal could have been potentially hindered by several yet-to-be resolved issues. The first issue is that there exists no clear guidelines for choosing species' similarity measures. For instance, the proportion of genes shared is seemingly a reasonable choice [15], but it is still ad hoc. The second issue is whether there exists enough information in the data to support a full-blown bifurcating hierarchy. It is reasonable to suspect that some parts of this full-blown tree structure are prone to be artifacts from the inherent features and assumptions of their model-based constructing approaches. The third issue is that there are irrelevant genes which should be taken off in the construction process. This issue becomes crucial when the number of involved genes is big. The last, but most essential, issue is that species phylogeny and genes' functional roles in speciation have not yet been connected. Indeed a phylogenetic tree is better perceived through the interacting patterns between species and genes clustering hierarchies. It is surprising to note that all these issues are different facets of the nonparametric information content of a binary bipartite network.

Nowadays such binary bipartite networks are ubiquitous in the sciences and real-world businesses. To cite a few here: boards of directors of companies [18], actors in movie [19], [20], antibiotic resistance vs. young calves [21], [22], antimicrobial resistance vs. rehabilitated northern elephant seals [23], and bacteria vs. flexible gene content of *Prochlorococcus* species [24]. Though the purposes behind these data collections, including the aforementioned ecological and phylogenetic ones, are very diverse, all these references also have commonly missed the critical information content in their binary bipartite network data sets.

In fact this phenomenon is detrimental to progress in scientific research and world businesses in general. Since, beside the network data, high-dimensional point cloud data have become the primary data type in this Internet and IT era. Any such a point cloud data set is naturally and apparently better perceived as a bipartite network because its sample size is just too small to sustain any smooth manifold or distributional structures under high dimensionality. For instance, a set of one billion 100-dimensional binary data points is merely a drop in the sea of its binary space 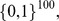 which has a cardinality of order 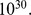 This is another major motivation why the nonparametric information content of a bipartite network is critical in building pertinent understanding under any high dimensional setting. Though the impact of the computational endeavors presented here are expected to be applicable to a wide spectrum of areas, we focus only on the above two real scientific examples for our expository purpose.

Before undertaking developments in sections below, we make clear a stand point that threads through all concepts, ideas and computations proposed in this paper. Our primary focuses are on issues such as “what is a pattern” and “how to recognize and compute patterns never seen before?”. These issues are not of classic statistics. In fact quantifying the notion of pattern and formalizing the process of pattern discovery in general are tasks in the heart of physical science [25]. Therefore when attempting issues as: what and where are patterns hidden within a binary bipartite network, we build a foundation such that a binary bipartite network is indeed a dynamics system. Such a physical stand is taken and emphasized in this paper. And it is because of this system stand point, this paper is never meant to be a statistical paper in classic sense. Further, as it is the case here, when an observed network is the only piece of data available to a scientist, he/she can not instantly invoke the classic concept of “sampling from a population”. Therefore classic statistical inferences could not be immediately concerned about without extra proper assumptions and supporting setups.

## Materials and Methods

### A Physical perspective: Where is the nonparametric information content?

In this paper we first give one physical perspective on the issue: What is the nonparametric information content? Denote an 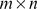 binary data matrix as 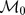, with spaces of row nodes 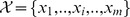 and of column nodes 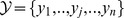. Let 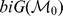 denote the binary bipartite network. This network is invariant with respect to the product of row and column permutation groups, denoted as 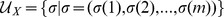 and 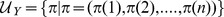, respectively. A permuted matrix is denoted as 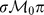. From the physical perspective, such a binary matrix 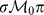 of 0 and 1 can be taken as an up-and-down spin configuration. Therefore the bipartite network 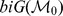 can be seen as the thermodynamic system defined by the collection of permuted matrices 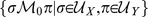 with Ising model potential [26]. Its ferromagnetic energy level is computed as follows:
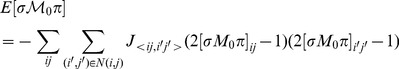
(1)where 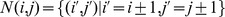 is the set consisting of the four nearest neighbors of the 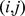 entry on the matrix lattice. Mirroring extensions are required for entries on the lattice edges, and the interaction potential 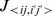 is taken to be constant 1 for simplicity. The negative constant on the left hand side of equation (1) defining the energy 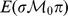 implies that aggregations of “up-spins”(1's) or “down-spins” (0's) on the field of 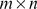 lattices tends to give rise to low energy levels, while spin configurations consisting of alternating 1's and 0's, as in a checker board, give rise to high energy levels.

This systemic concept of a bipartite network fits well in many biological settings. For instance, considering Darwin's finches and Case's Lizard data, their bipartite networks 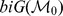 indeed globally approximate the interacting dynamics between species and islands along their evolutionary relational trajectories. Supposedly shaped by natural selection forces, the island-vs-species interacting patterns should be revealed in the information content of 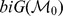.

The spin configuration achieving the lowest energy level is termed the ground state, or macrostate, of the system 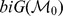. In statistical mechanics, a system's macrostate is supposed to reveal the most intrinsic behaviors and patterns of the system. Hence the macrostate is taken as the platform to manifest the coherent information content embedded within 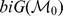. The computational complexity of finding such a macrostate is to solve for the minimizer 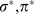 in the product permutation group 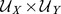:
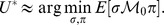



To resolving the discrete combinatorial optimization for 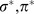, any direct search algorithm will encounter computational complexity when searching within a space of size 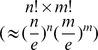 with 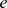 the Euler number. Due to this fact of exponential growth in size, it is nearly an impossible task even when 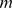 and 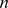 are only of moderate sizes. Instead of tackling this discrete combinatorial optimization with a direct optimizing approach as in statistical physics, we devise an indirect computing paradigm, called Data Mechanics, to resolve this computing issue in the next section.

### Data mechanics: A new computing paradigm

The principal theme of Data Mechanics is to divert the majority of computational complexity into engineering a data-driven surrogate system onto the product permutation group 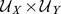. At least in an implicit fashion, this surrogate system is made to equip this group with a simple enough geometry such that its neighborhood system would not only allow us to avoid the majority of possible high energy spin configurations, but also allow the simple greedy search algorithm to reach the vicinity of the ground state.

**Figure 1:**
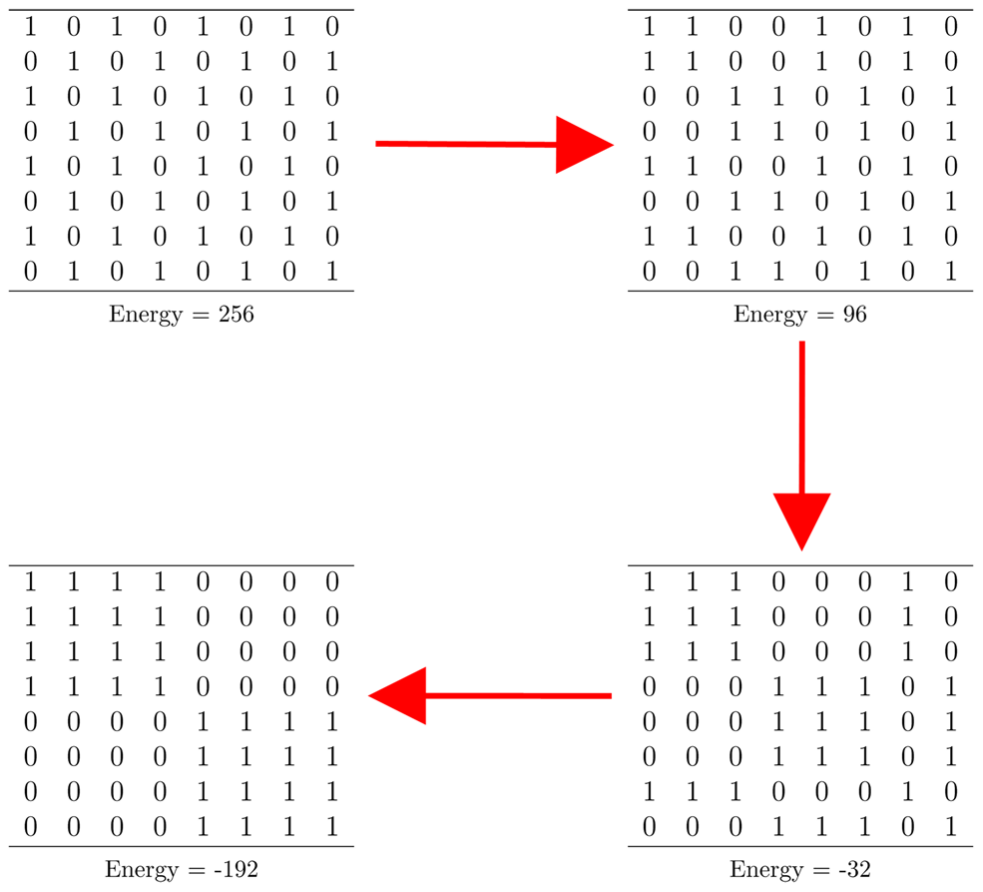
A schematic illustration of theme of Data Mechanics. Four 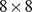 binary matrices are four different states of up-and-down spin configurations with their calculated energy levels. The upper left binary matrix with a checker board arrangement has the highest possible Ising model-based energy. By symmetrically grouping rows and columns, small scale blocks are formed first in the upper right matrix, and then larger blocks are seen in the lower right matrix, and then blocks of the global scale merge on the lower left matrix. The energy is successively reduced further down to the lowest one along this permuting process.

The possibility of this theme can be heuristically seen as follows. In order to avoid higher energy, first on the node scale level, we need to group similar rows and similar columns to form core clusters on 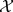 and 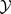, respectively. Since, by grouping similar columns, horizontal segments of 1's and 0's are created in the correspondingly permuted matrix 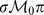. Likewise grouping similar rows generates vertical segments of 1's and 0's. Thus, by grouping rows and then columns or vice versa, many small blocks of 1's and 0's are generated on the lattice of 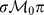. Further, on the core cluster scale, we apply the “similarity” idea again to facilitate merging of core clusters into conglomerate clusters on 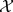 and 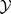, respectively. Consequently this joint operation would further reduce the Ising model-based energy due to emergence of larger blocks on a larger scale. We successively make use of the similarity idea on various scales to build a multiscale block structure, and expect to lower the energy level. In Fig. 1 we give a schematic illustration for this theme of Data Mechanics on a symmetry binary matrix example for simplicity.

In theory the permuting scheme illustrated in Fig. 1 should work equally well for binary bipartite networks and offers a glimpse of a natural geometry embedded within their ground states. However in reality it becomes extremely impractical to attempt to arrive at a macrostate of any real bipartite binary network via simply performing successive permutations. Typically after several permutations for grouping similar rows and columns, it seems difficult to proceed further beyond the node scale. The reason behind this difficulty is not only due to the lack of symmetry in a bipartite network, but more profoundly due to unknown patterns of interacting relationships between the two node spaces 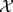 and 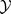. This real difficulty is clearly seen in the following real bipartite network data.

**Figure 2:**
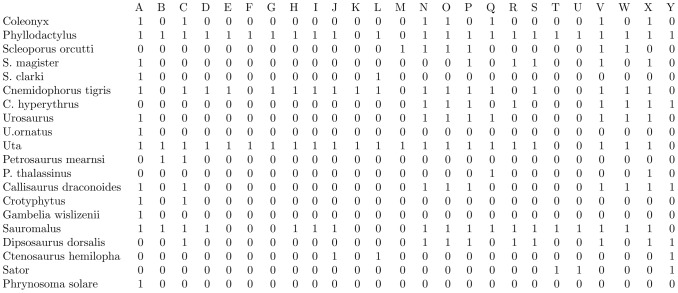
Case's (1983) Lizard data. 20 Lizard species vs 25 islands (in the Sea of Cortez (Gulf of California))

The Case's lizard data set [6] contains binary indices of 20 lizards' presence/absence on 25 islands, shown in Fig. 2. As a simple 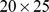 binary matrix, from the island aspect, the cardinality of the binary space 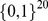 is over one million. If the 25 island data points are independently sampled from space 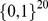 according to an unknown distribution, we expect to see a sparse and disconnected scattering of singletons. On the contrary, a composition of big or small clumps of data points is actually observed. This phenomenal data manifestation vividly indicates that the bipartite network data could have been a part of a “complex system” governed by highly structured rules and constraints. These rules and constraints, which are to be discovered from these network data, are parts of natural selection forces that governed the island-lizard interactions. Thus this phenomenal data composition of big or small clumps is envisioned in most real complex systems that give rise to large bipartite networks. In fact this complex system viewpoint is particularly necessary and true for big networks regarding the whole system of interest, such as the World-Wide Web or Facebook. Hence, in order to successfully discover unknown patterns of interacting relationships embedded within a bipartite network, we need an explicit algorithm to carry out the theme of Data Mechanics from the complex system perspective.

To motivate the construction for such an algorithm, we perceive the aforementioned phenomenal composition of big or small clumps from a geometric perspective and put forth an argument for adopting the ultrametric geometry. As similar enough data nodes form small clumps or core clusters, close enough core clusters merge into large clumps or conglomerate clusters. These various degrees of similarity and closeness are purely data-dependent. Thus, such a data-driven geometry would not only constitute the network's information content, but also would become a very effective tool for visualizing data with high dimensionality. The features of this computable geometry match with characteristics of an ultrametric geometry, that is, the whole composition can be specifically represented as a rooted “tree” in Graph theory.

An ultrametric is a metric, say 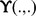, satisfying the strong triangular inequality,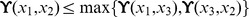 among any three nodes 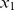, 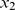 and 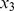. This property ensures any three points form either an equilateral or isosceles triangle. If we take a core cluster as a uniform component of point cloud data, then such a tree could be thought of as a geometric extension of Kolmogorov's algorithmic statistics with multiscale structural information. An informative review of ultrametrics and their biological applications is given in [27]. The discovery of ultrametrics and their implications in statistical mechanics is detailed in [28]. In recent computational geometry in mathematics, ultrametric spaces are studied from the perspective of embedding general metric spaces into trees [29], or reversely embedding trees into Euclidean spaces, called ultrametric skeletons [30]. A data-driven ultrametric for point cloud data or networks is algorithmically constructed based on devices taken from statistical mechanics [31].

A bipartite network involves two intrinsic geometries on the row and column axes, respectively. These two marginal geometries manifest through the same network data, so they must be closely interacting with each other. We collectively term all mutual interacting patterns “coupling geometry.” When the system's row and column node spaces consist of diverse groups of member nodes, it is natural that, as described and argued by the renown many-body physicist P. M. Anderson [32], the coupling geometry indeed involves interacting patterns, which likely merge via breaking-symmetry mechanisms in statistical physics. And, as put forth by H. Simon [33], the global architecture sustaining this coupling geometry must be a hierarchy. A hierarchy on the matrix lattice involved with underlying unknown breaking-symmetry mechanics is potentially to be a multiscale block structure, which induces two ultrametric tree-based marginal measures and satisfies the following information and mathematical criteria:

**[ET]**: Consider the finest scale clustering partitions on the two marginal trees as two discrete counting measures on row and column node spaces. The mutual entropy distances are sought to be as small as possible, while their individual entropies are as large as possible;

**[GW]**: The Gromov-Wasserstein distance [34] between the two ultrametric measure (u-mm) spaces is as small as possible.

Hence this coupling geometry is not only embraced in the minimum energy macrostate from a physical perspective, but also manifests their phenomenal interaction patterns with quantitative [ET] and [GW] features. The entropy feature [ET] requires marginal trees partitioning row and column nodes into as many core clusters as possible, and at the same time arranging these two clustering compositions into coherent association patterns, while the Gromov-Wasserstein distance feature [GW] requires the formation of a multiscale block structure throughout the matrix lattice. It is emphasized here that the coupling measure achieving the Gromov-Wasserstein distance can be mathematically thought of as a natural extension of the entropy-based mutual distance used in the [ET] feature to a multiscale geometric setting. Hence it provides a proper evaluation as well as a proper representation of the interacting relational patterns between two coupled measure-metric spaces. We further elaborate the functional merits of [ET] and [GW] features here, while their precise mathematical expressions are given in the next section.

The major merit of contemplating [ET] and [GW] features in a coupling geometry is that they jointly offer a computational foundation for constructing the theme of Data Mechanics, and at the same time shed the light for resolving the computational complexity of the discrete combinatorial optimization for searching the minimum energy macrostate. We devise an iterative data-driven computational algorithm to operationally build two tightly coupled ultrametrics in the latter section. As an ultrametric tree imposes strict constraints on node arrangements, the majority of nonviable permutations are in fact excluded. Further, the coupling of two marginal ultrametric geometries induces multiscale block patterns with contrasting high and low intensities of 1's or 0's onto the matrix lattice. A block with a high intensity of 1's (0's) indicates positive (negative) interaction between its row-cluster and its column-cluster. This kind of block pattern formation further reduces potential permutations on row and column because these blocks are to be moved around as unbreakable units under the two tree constraints in the process of minimizing energy levels. This characterizes how Data Mechanics resolves the computational complexity.

We illustrate our computational developments and coupling geometry via two real data sets: 1) 20 lizard vs. 25 islands [6]; 2) 8581 flexible genes vs. 12 species of *Prochlorococcus* and 4 species of their close relatives, *Synechococcus* [24]. The Lizard-island data set can also be found in Table 1 of [9], while the second data set can be downloaded from Table S1 of [24] at doi:10.1371/journal.pgen.0030231.st001.

### Algorithm in Data Mechanics

Now we propose our critical algorithmic construction for the pair of ultrametrics 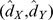 that is supposedly approximating the optimal pair 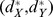 embedded within 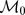. As mentioned in the previous section, an ultrametric is typically transformed from an empirical measure. In this paper such a transformation is proposed by applying the Data Cloud Geometry (DCG) algorithm developed in [31]. In general via subject matter knowledge, we choose an empirical distance (or similarity) measure 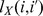 for any node-pair 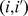 from 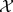 and 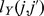 for any node-pair 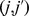 from 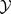. Here the initial choices for both 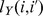 or 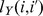 are the Hamming distance.

Without loss of generality, suppose that the number of rows 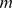 is smaller than the number of columns 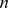. Based on the initial choice of 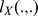, the DCG algorithm is applied to build an ultrametric tree as an initial version of the target 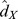 on 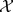. Denote the ultrametric tree distance matrix among all nodes in 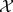 as 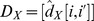, where 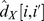 is the ultrametric distance between 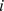 and 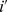 row-nodes.

The use of 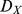 is to provide a geometric basis for defining a modified version of the Hamming distance as an initially deduced empirical measure 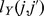 on 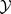. One version of 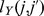 on 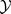 deduced from 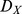 is constructed as follows: Let 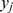 and 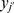 be any pair of column vectors in the space 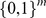. For a discordance at the 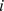
th component 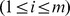, say 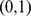, between 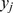 and 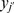, (that is, 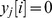 and 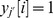), we consider the collection 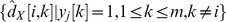. This collection shows the “anti-potentials” of co-presence of the 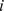
th “feature (being present)” with those existing “features” in 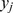. Denote the median of this collection as 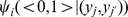. Similarly we define the 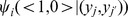. We then define
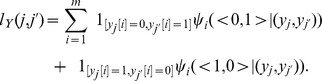



In contrast the Hamming distance between 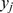 and 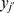 is 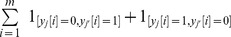. Thus this measure 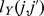 on the column vectors incorporates the geometric distinction among the 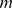 components based on 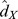, to which the Hamming distance does not.

The next step is to apply the DCG algorithm based on the deduced 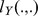 to build an ultrametric tree on 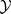. This is an initial version of the target 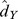. Denote the ultrametric tree distance matrix among all nodes in 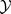 as 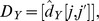 where 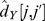 is the ultrametric distance between the 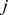 and 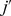 column-nodes. Then we modify the initial version empirical measure 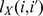 on 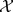 based on 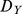 via the same deducing procedure as above. Iterative modification is performed back and forth between these two node-spaces 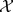 and 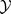 until the pair of ultrametric trees 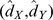 becomes stable.

The key concept behind this iterative algorithm is to bring out the circular relationship between the inter-dependence patterns contained within the bipartite network 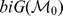 and its two marginal ultrametric spaces 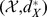 and 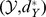 in a data-driven fashion. Therefore, by implementing this iterative algorithm, we anticipate phenomenal multiscale block structural information being revealed as features of the coupling geometry. This algorithm is applied on two illustrating examples experimentally to provide evidence that our iterative approach is capable of discovering a pair of (nearly) optimal ultrametrics 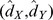 from 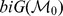.

## Results

### Community ecology example

**Figure 3:**
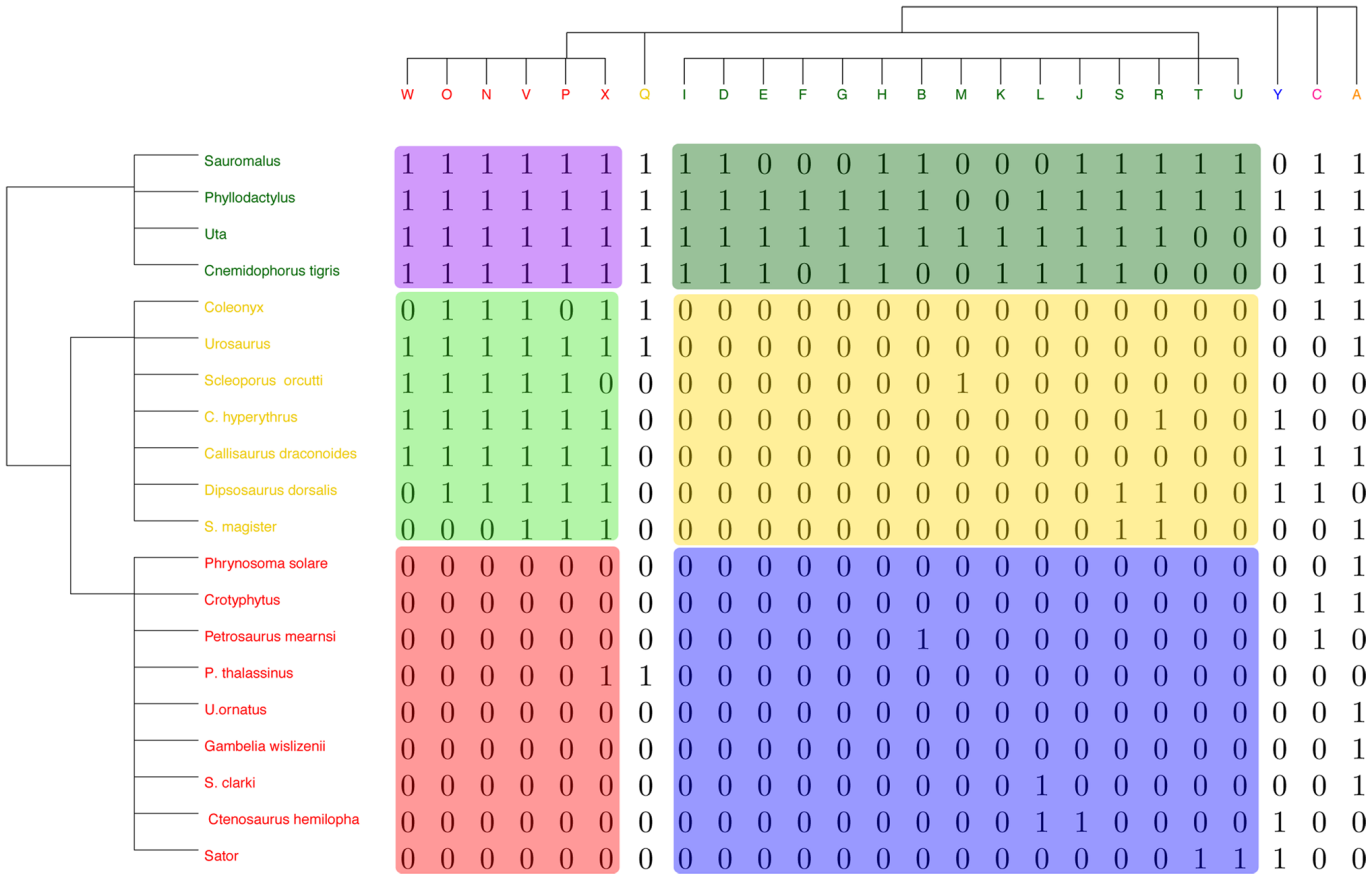
The computed coupling geometry of Case's lizard data with marked blocks for interactions. Two marginal ultrametric trees are shown on row and column space. The initial and final energy levels are −748 and −1426.

Consider the presence/absence data matrix of lizard vs. island in [6] (see also [12]), as shown in Fig. 2. We apply the above iterative algorithm and arrive at a pair of stable distance measures on both spaces of row and column nodes, respectively. The coupling of these two computed ultrametrics reveals structural block patterns, as shown in Fig. 3. It is noted that the coupling geometry has been obtained by permuting the blocks and then permuting nodes within each block to achieve further reductions in energy level.

The coupling geometry shown in Fig. 3 clearly reveals that the marginal geometry on row axis has three clusters with two of them being closer than either one of them to the third cluster, while another marginal geometry on the column axis essentially has two major clusters and three singletons as outliers. The coupling geometry allows us to visualize one cluster of species as being the least adaptive by having negative interactions with most of the islands, except with those three outlying islands. In contrast, the other cluster of species is the most adaptive group throughout all islands, while the third cluster of species' adaptation is selective on island clusters. The vividly contrasting interacting patterns in this coupling geometry should provide rather informative evidence for discerning and testing community assembly rules. Specifically, computed geometries based on species' and island's covariate information must conform to this coupling geometry. This is an example of the implied global inference, which is contrastingly different from Monte Carlo based statistical inference in the literature. Most of the popular Monte Carlo schemes are based on uniform distributions of the degree sequences. They are not even related to the marginal measures 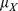 or 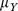. Thus they typically miss the system perspective of the coupling geometry.

Next we conduct a computer experiment to confirm that our computed coupling geometry is indeed in the vicinity of the ground state from the physical perspective. This experiment consists of a simple local perturbation, called checkerboard-switch, for generating new binary matrices. A checker-board is a 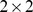 binary matrix with all row and column sums being equal to 1. We say such a matrix is in A-state if both 1's are on the main diagonal, and B-state for off-diagonal ones. A switch is to change A-state into B-state, or vice versa. Such a checkerboard-switch was proposed in [6] and popularly used in the literature of community ecology on assembly rule (see also [12]).

**Figure 4:**
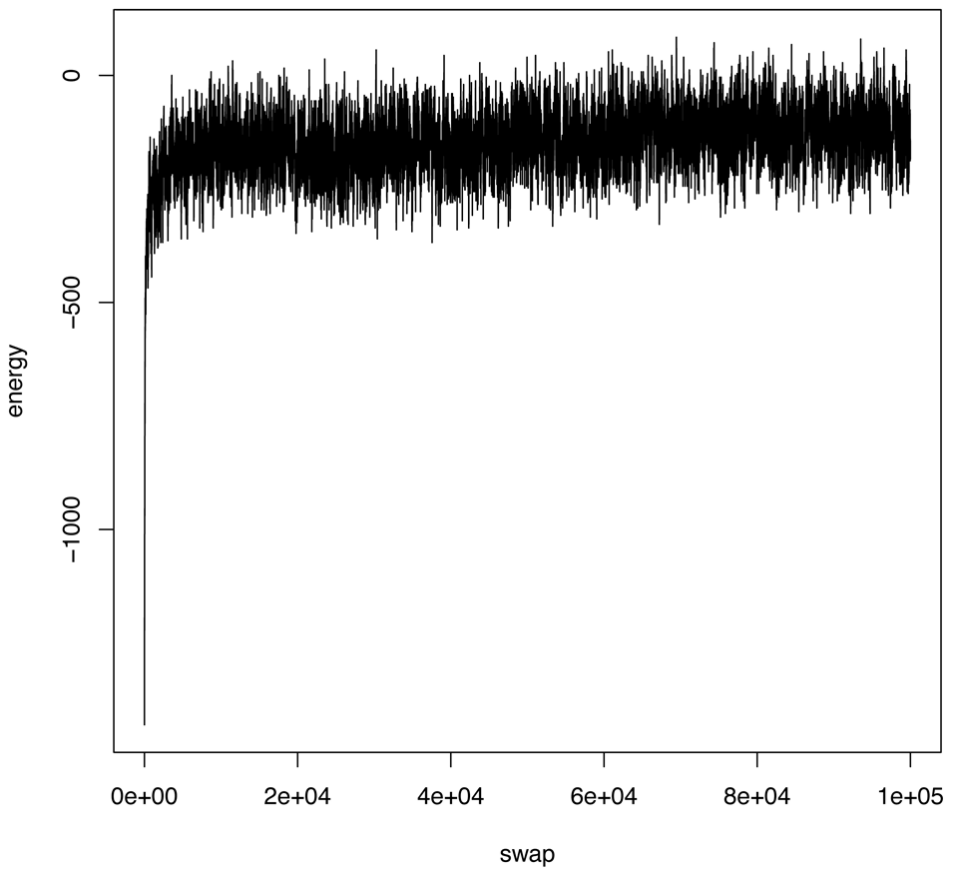
Evidence of macrostate of the computed coupling geometry of Case's lizard data. The energy levels from a series of matrices are reported. The matrices are generated by randomly switching a checkerboard in the last matrix.

This experiment begins with randomly choosing two rows and two columns from the computed coupling geometry shown in Fig. 3. If the four entries do not form a checkerboard, then make another run of random selection. If it is a checkerboard, then a switch is performed, and resulting in a locally perturbed new 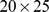 matrix. Its energy level is computed. This is the first trial. The next trial implements the random checker-board switch perturbation onto the matrix resulting from the previous trial. We repeatedly continue this perturbation process for 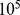 trials, and then plot the sequential 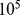 energy levels, as shown in Fig. 4.

The energy trajectory makes a nearly vertical jump from the energy value at 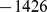, then successively goes through a plateau and then somehow is trapped in a level-off phase, in which it oscillates between −200 to −100. This distinct phenomenon reveals the fact that this checkerboard-switched matrix sequence escapes extremely quickly from a deep potential well, which is confirmed as a ground state. In fact, the level-off phase in Fig. 4 is not an absorbing phase. The trajectory eventually evolves into another phase with energy level near 0 when this experiment continues beyond several millions trials. Though, at this stage, we have no exact knowledge about how many leveling-off phases are actually involved in this experiment, we imagine that the number is closely tied to the multiscale block patterns embraced within the coupling geometry.

Nonetheless this energy trajectory clearly demonstrates that the computed coupling geometry is at least very close to the minimum energy macrostate from the physical perspective. Also the clear block pattern information in Fig. 3 simultaneously provides evidence for achieving the optimal coupling measure regarding the [ET] and [GW] features from a mathematical perspective. Hence we are confident to summarize that our Data Mechanics and its algorithm successfully achieve its designated task of extracting nonparametric information content embedded within this data set.

### Phylogeny based on gene content

Next we illustrate a bipartite network in phylogeny. The advent of completely sequenced genomes has provided a new perspective for constructing phylogeny based on gene content. Via a bipartite network, this new perspective offers that phylogeny is better viewed as systemic interactions between genes and species in order to capture the evolutionary trajectories of gain-and-loss on genes. Such a gene-content-based phylogeny is expected to be less sensitive to inconsistencies due to horizontal gene transfer and highly variable rates of evolution, to which single gene based phylogeny, in general, suffers.

Consider a data matrix taken from [24] with 8581 flexible genes on 12 species of *Prochlorococcus* (6 for high-light(HL) and 6 low-light(LL)) and 4 species of its close relative, *Synechococcus*. These 8581 flexible genes contain only 481 distinct combinations of a gene's presence/absence in the 16-dim binary space 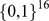. Among these 481 genes, a giant clique of size more than 300 is formed according to the Hamming distance (see Figure S1(a) in File S1). The presence of this giant clique strongly indicates that these 481 genes are indeed selected via a highly structural scheme. In contrast, when 481 genes are indeed independently sampled from the 16-dim binary space 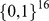, the chance of finding a clique of size more than 3 is negligible (see File S1 and Fig. S1(b) in File S1). We begin our computing by first tentatively trimming off non-informative genes in distinguishing among species, such as genes with less than 4 and more than 14 presences among the 16 dimensions. There are 312 remaining genes. We then apply the iterative algorithm on this 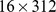 binary matrix.

**Figure 5:**
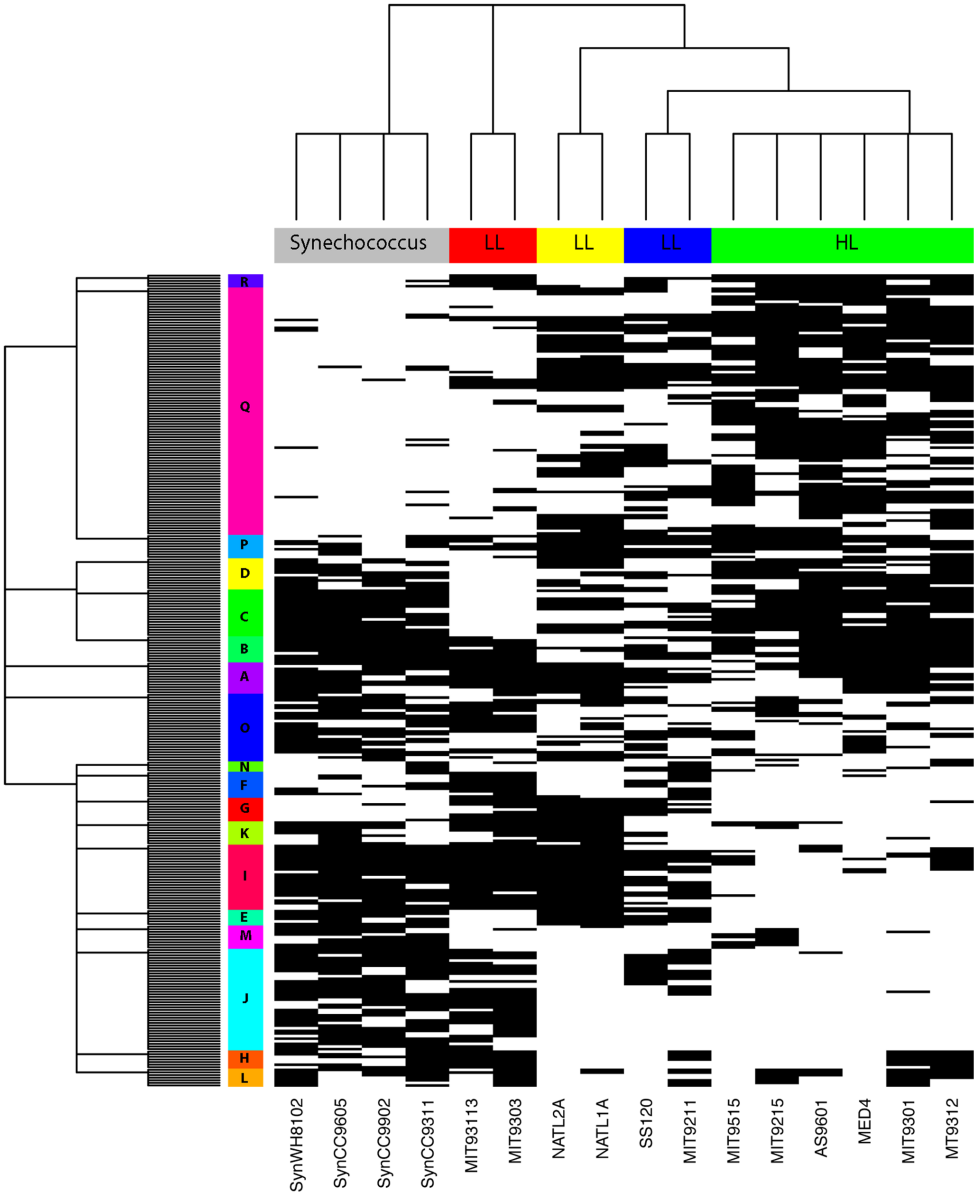
The computed coupling geometry of 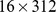 bipartite presence/absence matrix of gene content. Phylogenetic tree of 12 species of *Prochlorococcus* and 4 species of *Synechococcus* and a gene tree of 312 distinct genes are shown on the row and column space. The initial and final energy levels are −7524 and −10880.

The coupling geometry, shown in Fig. 5, is obtained by schematically permuting blocks and then nodes within blocks. All permutations are selected to achieve lower energy levels, while conforming to the two marginal tree geometries: a phylogenetic tree on the 16 species and a gene-tree equipped with 18 core clusters. It is rather interesting to see that our nonparametric species phylogenetic tree is characteristically similar with those model-based trees reported in [24]. From the coupling geometry, the gene contents of the two far apart core clusters: 6 HL *Prochlorococcus* and 4 *Synechococcus* species, nearly complement each other. They overlap on three small gene clusters: B, C and D, which jointly form a single branch in the gene tree. Contrastingly the 6 LL *Prochlorococcus* species are divided into three pairs, each of which has rather distinct gene content. A pair (in red) is indeed so different that it becomes an independent branch in the phylogenetic tree. This geometric feature is not found in the classic phylogenetic tree, which is mostly bifurcated. In fact their gene content vividly support this data-driven tree geometric structure. As for the gene tree, 16 out of 18 gene core clusters reveal their speciation functions, except the two irrelevant gene clusters A and O. In summary this coupling geometry, which should be in the close vicinity of the minimum energy level, is rather informative for gene-vs-species interaction. It is also worth noting that this computing process also resolves the issue of how to identify irrelevant feature dimensions.

### Bootstrapping and statistical implications

One significant merit of the coupling geometry is that it serves as a foundation for the principle of bipartite network bootstrapping. This systemic principle is prescribed as follows:

#### Principle of Bipartite Network Bootstrapping

All bootstrapped networks of a bipartite network 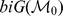 are generated as microstates conforming to the macrostate.

Here the macrostate is the computed coupling geometry via 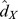 and 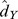 as an approximate for 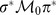. By “conforming” we mean that the block structural information of the finest scale has to be retained in every bootstrapped bipartite network. Therefore a bootstrapped bipartite network must be constructed by pitching up all simulated blocks subject to row and column sums of the original sub-matrices. This principle brings out an essential and relevant fact that any bootstrapping ensemble has its corresponding structural constraints. Any of its member should not be treated as a random graph even though it certainly contains randomness within blocks. But more importantly it also embraces deterministic structures between blocks. This characteristic of simultaneously embracing deterministic structures and randomness is a defining feature of real dynamics systems [25].

For implementing the network bootstrapping principle here, an algorithm proposed in [35] is suitable for this block-wise simulation (also see a modified version in [36]). We give the modified algorithm below for generating a submatrix with given row and column sum sequences.

#### Algorithm for random submatrix with given row and column sum sequences

Denote the sub-matrix as 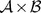 with the two node subspaces 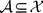 and 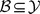. Denote the row sums, or degree sequence 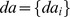 of 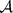 and column sums, or degree sequence 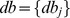 of 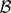. Let 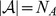 and 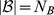.

**Initialization**: Set 
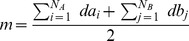
. Initialize the set of edges 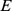 to the empty set. Define the sequences 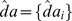, 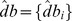 and initialize it by 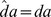 and 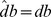. Set 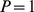.

**Step 1**: Pick one node pair 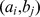, 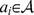 and 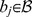, with probability proportional to
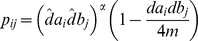
among all pairs 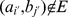.

**Step 2**: Update 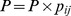, and add 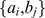 to E and reduce 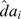 and 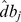 by 1.

**Step 3**: Repeat Step 1 and Step 2 until no more edges can be added to E.

**Output**: If 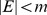 report failure, otherwise output 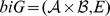 and 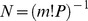.

Here the number 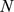 returned by the algorithm is an estimate of the size of the ensemble of corresponding random graphs. Therefore the size of a bootstrapping ensemble of the whole bipartite network is calculated as the product of the sizes of all block ensembles.

It is noted that the original version of the algorithm proposed in [35] has the probability of picking a pair of nodes 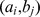 with the exponent 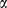 being equal to 1. We noticed however that when one or multiple hubs are present in the block considered, the algorithm often fails to generate a network that fully satisfies the constraints on the degrees of the nodes. To resolve this problem, we define:
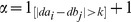
(2)that is, 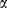 is an indicator function with an empirically chosen thresholding exponent 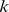 (i.e. 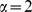 if 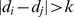 and 1 otherwise). This is a pragmatic correction that circumvents the failure problem, with the side effect of underestimating the size of the corresponding ensemble. Also we note that there are other algorithms described in the literature for generating binary matrices with prescribed row and column sums (see for example [1]). These algorithms rely on the maximum entropy property and provide asymptotic estimates of the size of the ensemble of random networks that can be generated. The algorithm used here has the advantage of being simple to implement.

It becomes evident that, under this principle of bipartite network bootstrapping, any bootstrapped bipartite network is equipped with the characteristic multiscale block pattern information. Here we further explore such a multiscale block structure from the energy distribution perspective. Consider bootstrapping the Lizard's bipartite network data based on the computed coupling geometry shown in Fig. 3. We construct three different bootstrapping ensembles with respect to three different scales of block structures as follows:

**En-1** The finest scale level: the 6 colored blocks in Fig. 3 are simulated individually with the remaining four outlier columns (A, C, Y, Q) fixed; The estimated logrithm of this ensemble size is 25.68.

**En-2** One median scale on row axis: the green and orange blocks are merged into one larger block, or submatrix, and yellow and blue blocks are merged into another larger submatrix; The estimated logrithm of this ensemble size is 28.43.

**En-3** One coarse scale on row and column axes: the four blocks marked with colors: green, orange, yellow and blue, are merged into one large submatrix. The estimated logrithm of this ensemble size is 136.76.

**Figure 6:**
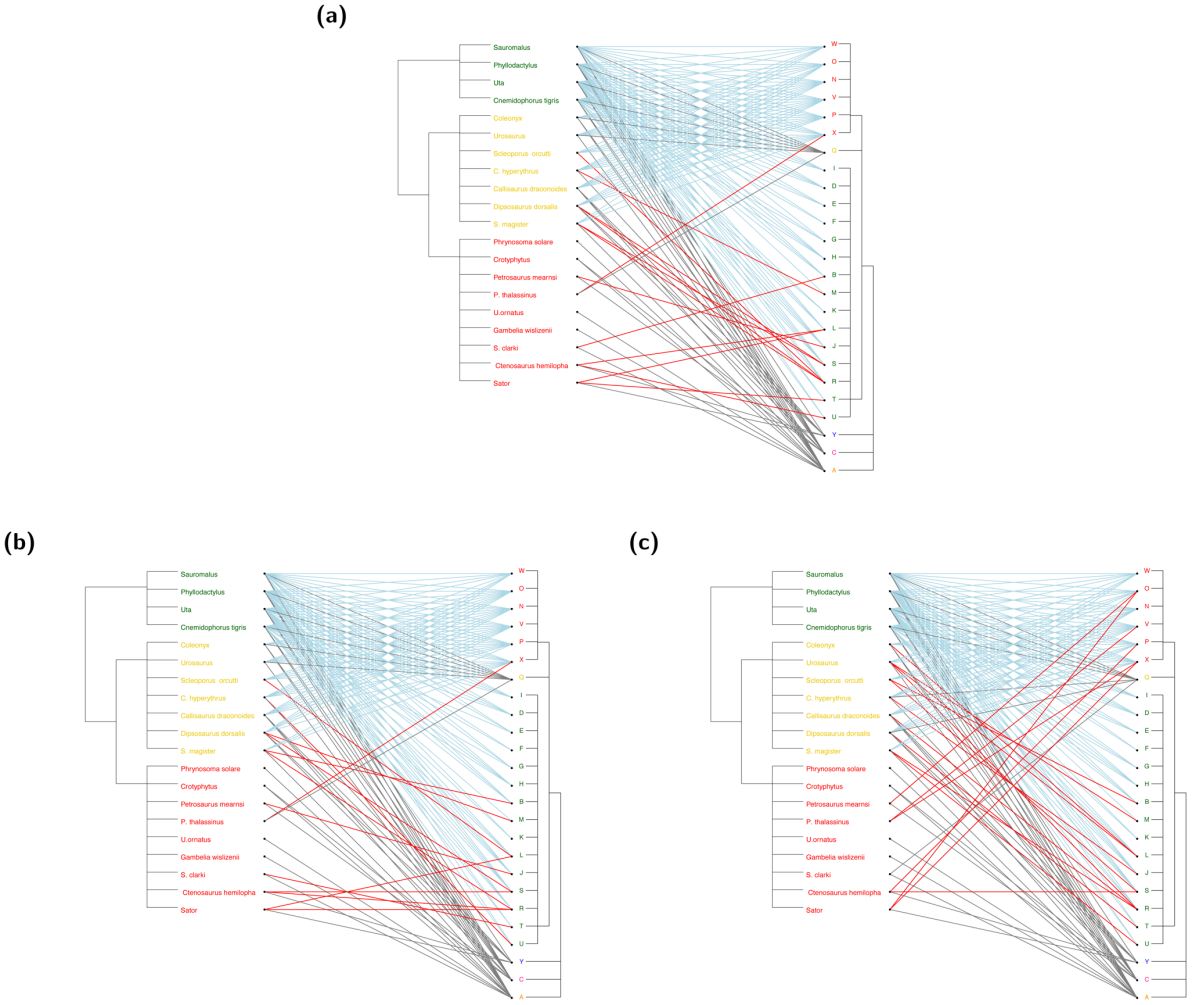
Three bootstrapped bipartite networks from En-1 (a), En-2 (b) and En-3 (c) bootstrapping ensembles based on the computed coupling geometry of Case's lizard data. In contrast to the presence-absence matrix in Fig. 3, more presence connections (1's in the matrix) are observed in the yellow, blue and red blocks as the one-scale structure is relaxed. These connections are marked in red.

**Figure 7:**
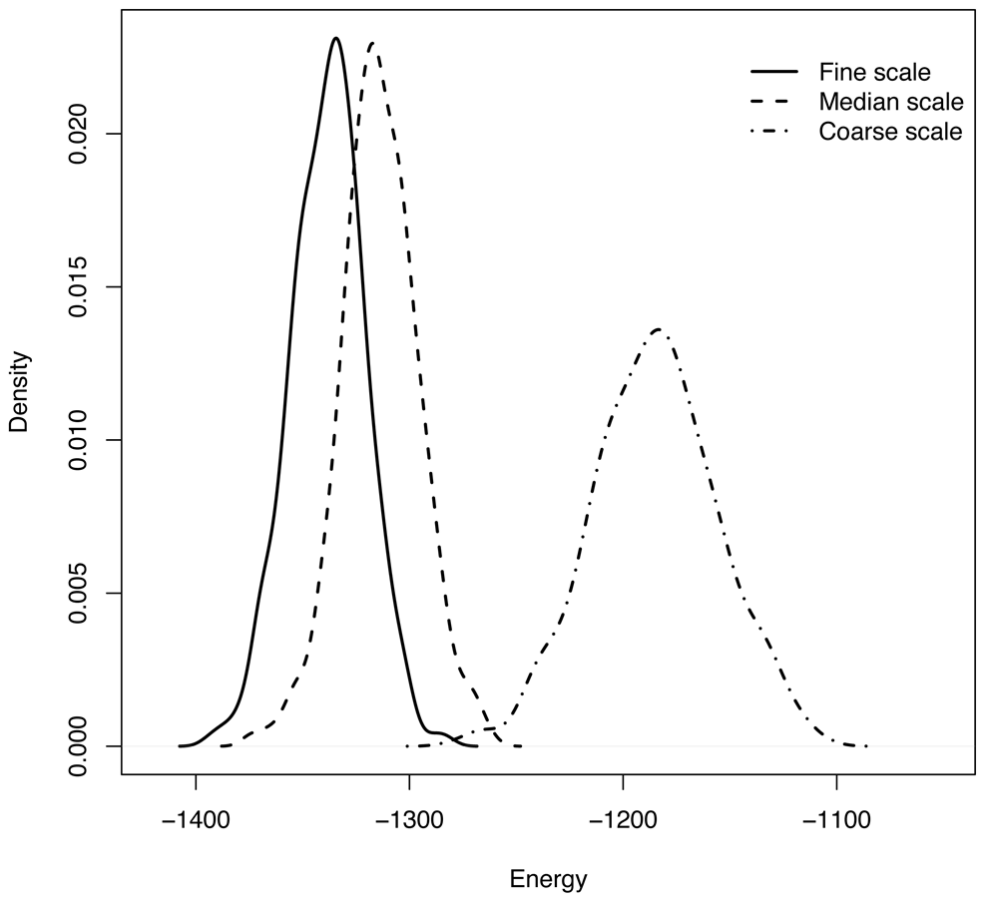
Three energy density functions from En-1, En-2 and En-3 bootstrapping ensembles. The three energy densities from left to right correspond to block structures En-1, En-2 and En-3 based on the computed coupling geometry of Case's lizard data, as shown in Fig. 3.

We randomly select one bootstrapped network from each of the three ensembles, En-1, En-2 and En-3, respectively, and present them in Fig. 6. The first two bootstrapped bipartite networks are hardly different, and the third one is only slightly different from the first two. Correspondingly the energy densities of these three bootstrapping ensembles with respect to block structures En-1, En-2 and En-3 are reported in Fig. 7. The first location shift from the very left density of En-1 to the middle density of En-2 is relatively small. It indicates that the merging of green and orange blocks together with merging of yellow and blue blocks do not create significantly different matrices. This indication is reasonable and real because one of the four involved blocks is nearly completely 1's, while the rest of three blocks are nearly completely 0's. No significant different matrices are to be expected. In contrast, the second location shift of the middle density to the very right one of En-3 is relatively larger than the first location shift. The merging of the four blocks: green, orange, yellow and blue, allows us to create more distinct matrices, but not drastically different. In fact these four blocks form an “AND” type of interacting pattern, in contrast with the “OR” interacting pattern shown in Fig. 1. We expect a very large bootstrapping ensemble only when the 6 colored blocks are merged.

Our computed coupling geometry manifests data-driven features, so it is likely biologically meaningful. Upon its multiscale block structural information, different scales can bear with different biological features of interest. The presence of this fine scale feature is meant to contain complicated and detailed structural information, while the presence of only large-scale features is meant to lose all fine-scale structural characteristics. Thus along the multiscale, scientists can formulate a serial of nested null/alternative hypotheses. Then, based on the corresponding bootstrapping ensembles, proper bootstrapped distributions of testing statistics can be constructed. Making comparisons among such bootstrapped distributions is a legitimate way of making statistical inferences. Another natural testing statistic is the ratio of the sizes of the bootstrapping ensembles. Such a ratio reveals the probability of seeing a network equipped with structural patterns specified by an alternative hypothesis within a larger ensemble consisting of bootstrapped networks lacking the specified patterns under the null hypothesis.

In network applications, the most popularly employed null hypothesis corresponds to the largest possible scale level, which usually lacks any block structure. Its bootstrapping ensemble in turn contains patternless networks that are only subject to the full spectrum of row and column sum sequences. This largest ensemble surely contains networks having relatively smaller-scale block pattern information as its minority and patternless networks as its majority. It is worth noting that, due to its massive size, it might require a very large number of bootstrapped networks in order to build a representative bootstrapping distribution for any testing statistics.

On the other hand inferential issues on hypotheses concerning features beyond the computed pattern geometry can not be immediately and rigorously discussed. The following reason is one of the keys: as far as model based statistical inferences on networks are concerned, they are likely imposed with many men-made and unrealistic structural assumptions. Also the model setups also tend to involve with many parameters. The implication deduced from this paper is that these assumptions and parametric setups often are incoherent, or even contradicting with the computed geometric information. Such incoherence and contradiction would fundamentally cause many difficulties. It is easy to speculate that a complex network model is built without a solid capability of accommodating potential structural information contained in the data-driven macrostate. Hence, not only computational, but also inferential issues could spin out of control. These related phenomena are of critical importance. However they are beyond the scope of the current paper.

## Discussion

The information content of a binary bipartite network is identified, computed and represented as a coupling geometry. From a physical perspective, it is a pure combinatorial optimization problem with rather overwhelming computational complexity. Our Data Mechanics is proposed as an indirect optimization approach to effectively resolve such a complexity. Through information entropy criteria and the Gromov-Wasserstein distance, the coupling geometry is found to be equivalent to an optimal construction problem for a pair of ultrametric measure spaces. In addition, the computed coupling geometry is capable of manifesting authentic interacting patterns on different layers of geometric hierarchies.

The resultant geometric multiscale pattern information is demonstrated to be able to shed new light on two important biological topics: 1) species and island interaction in community ecology; 2) phylogeny based on gene content in genetics. In principle this coupling geometry would serve as a foundation for global inference. For instance, the bipartite network of island and species can be taken as a response, and bipartite networks of islands' and species' individual information as a covariate. Coupling these three geometries would fundamentally resolve the controversial community assembly rules. Potential applications of such global inferences are ubiquitous in science.

Beyond shedding new and critical light on real world problems, the coupling geometry and Data Mechanics together seems to point out an important cycle among three elements: a binary data matrix naturally contains an optimal pair of ultrametrics 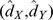; such an ultrametric pair supports a coupling geometry; a coupling geometry then reveals the authentic interacting pattern information content embedded within this data matrix. This trinity concept implies one principle for network analysis in general: a computable coupling geometry is the foundation for legitimate statistical modeling and hypotheses formulating, while such geometry based network bootstrapping ensembles provide the bases for hypothesis testing and statistical inference. That is, computing geometric information content from matrix data is a principle way of seeking knowledge from networks ranging from binary to weighted, undirected to directed.

## Supporting Information

File S1 (PDF)
