# Supplementary material for: Data Mechanics and Coupling Geometry on Binary Bipartite Networks
Source: PLoS One. 2014 Aug 29;9(8):e106154. doi: 10.1371/journal.pone.0106154 (PMC4149528; doi:10.1371/journal.pone.0106154)
Supplement: File S1 — (PDF) [file pone.0106154.s001.pdf]

Supplementary File S1 for

Data Mechanics and coupling geometry on binary bipartite networks

Hsieh Fushing*and Chen Chen

phylogeny based on gene content

In this supplement we first report that the 8581 flexible genes have been selected via a high-degree "non-random" scheme, and then show that several subgroups of genes indeed have rather sharp distinguishing capability among the three groups species: High-Light Prochlorococcus (HL), Low-Light Prochlorococcus (LL), and Synechococcus (Syn).

Consider the space $\{0,1\}^{16}$ of all 16-dim binary vector-nodes equipped with Hamming distance, which counts the number of discordant pairs of vector components, a simple network the space is constructed by linking two vector-nodes with an edge if the Hamming distance between them is equal to one. Such a connectivity gives rise to several simple facts as that, among the $2^{16}(> 64 \times 10^3)$ nodes in $\{0,1\}^{16}$, each vector-node is k distance away from $C_k^{16} = \frac{16!}{k!(16-k)!}$ many vector-nodes for all $k = 1, ..., 16$. Therefore the 2^{16} vector-nodes form a homogeneously connected graph with constant degree 16 for all nodes. This graph is denoted as $G_H[2^{16}]$. The homogeneity indicates that each vector is at the same neighboring relations as all the others have, so there is no clear geometric structure on space $\{0,1\}^{16}$ under Hamming distance. On the other hand, it is clear to image that if the majority of vector-nodes are completely randomly removed from $G_H[2^{16}]$ along with the edges connecting to them, the remaining graph would fall into many isolated pieces.

Back to the phylogeny data set, among the 8581 genes, there are 481 distinct 16-dim binary vectors. Consider the subgraph of $G_H[2^{16}]$ with these 481 vector-nodes and edges pertaining to them. We term this subgraph $G_H[481]$. Suppose that the $(2^{16} - 481)$ vector-nodes are completely randomly removed from $G_H[2^{16}]$, in other words, 481 vector-nodes are randomly selected with their connectivity, $G_H[481]$ should be highly disconnected in a sense of having only cliques of rather small in sizes. This is because the probability of randomly selecting a 16-dim binary vector with Hamming distance being equal to one to the given vector is $16/2^{16}$, which is less than 0.0002. However, countering this expectation, the real network $G_H[481]$ retains a giant clique of size 379. A hierarchical clustering tree of these 481 vector-nodes with Hamming distance via single linkage is shown in Fig. S1(a).

This simple analysis reveals that the selection of the 8581 genes must have been involved with a highly structural mechanism, so that the presence of such a giant clique in $G_H[481]$ is possible. To support this statement, we randomly simulate a network with 481 vector-nodes as illustration, denoted as $\hat{G}_H^S[481]$ with uniform probability on $\{0,1\}^{16}$. A hierarchical clustering tree of $\hat{G}_H^S[481]$ reveals contrasting and striking differences by having no cliques of size more than 3, as shown in Fig. S1(b).

*Department of Statistics, University of California, Davis

Given that the network $G_H[481]$ does not afford clear structural information among 481 distinct vectors, the giant clique seems to offer an interesting base to look further into. We propose breaking the giant clique into pieces, and then looking into each sizeable pieces. To do so, we empirically further remove all vector-nodes with total presences being less than 4 or larger than 13 among the 481 vectors for not being informative in differentiating species. The removal scheme results into 312 vector-nodes, containing 286 vector-nodes from the giant clique. The resultant hierarchical clustering tree is shown as Fig. S1(c). The tree level at distance 1 (the bottom level) reveals 3 large cliques (marked from A through C) and three smaller-sized ones. It is not surprising that each of these six cliques reveal rather strong, but distinctly visible characteristics that can differentiate between the three species groups: HL, LL, and Syn.

These gene cliques with differentiability on the three species groups: HL, LL, and Syn, bring out the essential aspect of why constructing a realistic geometry on gene dimension is crucial for phylogeny. In order to make the gene-species relational coupling geometry even more explicit, we elaborate as follows. With respect to all genes, each species' gene content is a 8581-dim vector (or 312-dim vector if only counting for genes with distinct and informative gene content). By using Hamming distances as the initial distance measure, the 16-species vectors will give rise to a distance matrix with which an initial phylogenetic tree is derived by DCG algorithm. A modified version of Hamming distance is then derived for gene's 16-dim vectors, and a DCG-based ultrametric tree on genes is derived. In this fashion, the iterative procedure is implemented to derive the data-driven distance measures for the species and genes. The resultant trees for species and genes would accordingly rearrange the matrix of gene content (i.e. presence/absence of genes) to reveal multiscale block structural information. Therefore a phylogenetic tree is not the only goal in phylogeny. The ultimate goal is the coupling geometry constituted by the genes vs. species relational patterns. Ideally we obtain information of which gene clusters play which roles in differentiating among species groups HL, LL, and Syn, and which gene core clusters can differentiate species within the three species groups. Therefore it is extremely important that we can systematically derive such gene vs. species relational coupling geometry. On top of the fact that our coupling geometry based phylogeny avoids unrealistic assumptions on speciation, this geometry offer an advantageous platform for identifying functional roles of gene-clusters in phylogeny.

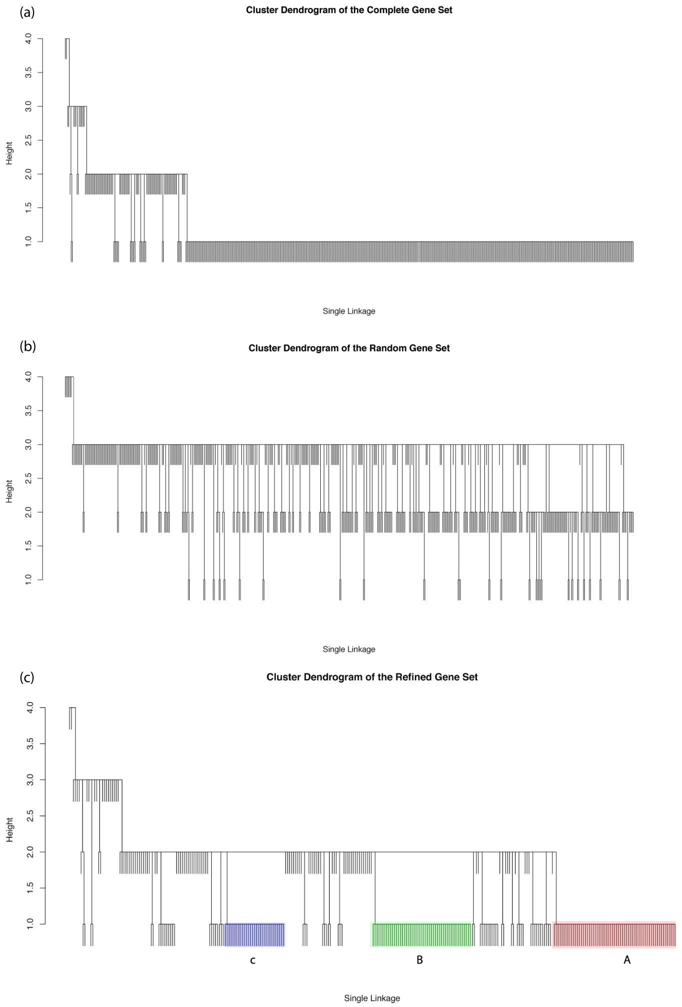

Figure S1: Exploratory hierarchical clustering tree with Hamming distance and single linkage.
